# A Unified Spatiotemporal Modeling Approach for Predicting Concentrations of Multiple Air Pollutants in the Multi-Ethnic Study of Atherosclerosis and Air Pollution

**DOI:** 10.1289/ehp.1408145

**Published:** 2014-11-14

**Authors:** Joshua P. Keller, Casey Olives, Sun-Young Kim, Lianne Sheppard, Paul D. Sampson, Adam A. Szpiro, Assaf P. Oron, Johan Lindström, Sverre Vedal, Joel D. Kaufman

**Affiliations:** 1 Department of Biostatistics, and; 2 Department of Environmental and Occupational Health Sciences, University of Washington, Seattle, Washington, USA; 3 Institute of Health and Environment, Seoul National University, Seoul, Korea; 4 Department of Statistics, University of Washington, Seattle, Washington, USA; 5 Core for Biomedical Statistics, Seattle Children’s Hospital, Seattle, Washington, USA; 6 Centre for Mathematical Science, Lund University, Lund, Sweden

## Abstract

**Background:**

Cohort studies of the relationship between air pollution exposure and chronic health effects require predictions of exposure over long periods of time.

**Objectives:**

We developed a unified modeling approach for predicting fine particulate matter, nitrogen dioxide, oxides of nitrogen, and black carbon (as measured by light absorption coefficient) in six U.S. metropolitan regions from 1999 through early 2012 as part of the Multi-Ethnic Study of Atherosclerosis and Air Pollution (MESA Air).

**Methods:**

We obtained monitoring data from regulatory networks and supplemented those data with study-specific measurements collected from MESA Air community locations and participants’ homes. In each region, we applied a spatiotemporal model that included a long-term spatial mean, time trends with spatially varying coefficients, and a spatiotemporal residual. The mean structure was derived from a large set of geographic covariates that was reduced using partial least-squares regression. We estimated time trends from observed time series and used spatial smoothing methods to borrow strength between observations.

**Results:**

Prediction accuracy was high for most models, with cross-validation *R*^2^ (*R*^2^*_CV_*) > 0.80 at regulatory and fixed sites for most regions and pollutants. At home sites, overall *R*^2^*_CV_* ranged from 0.45 to 0.92, and temporally adjusted *R*^2^*_CV_* ranged from 0.23 to 0.92.

**Conclusions:**

This novel spatiotemporal modeling approach provides accurate fine-scale predictions in multiple regions for four pollutants. We have generated participant-specific predictions for MESA Air to investigate health effects of long-term air pollution exposures. These successes highlight modeling advances that can be adopted more widely in modern cohort studies.

**Citation:**

Keller JP, Olives C, Kim SY, Sheppard L, Sampson PD, Szpiro AA, Oron AP, Lindström J, Vedal S, Kaufman JD. 2015. A unified spatiotemporal modeling approach for predicting concentrations of multiple air pollutants in the Multi-Ethnic Study of Atherosclerosis and Air Pollution. Environ Health Perspect 123:301–309; http://dx.doi.org/10.1289/ehp.1408145

## Introduction

Cohort studies have shown an association between long-term exposure to air pollution and cardiovascular morbidity and mortality (Brook et al. 2010; Miller et al. 2007; Pope and Dockery 2006). To estimate these associations, epidemiologic studies develop exposure prediction models to predict pollutant concentrations over long periods of time at cohort home addresses based on monitoring data from regulatory networks or study-specific monitoring campaigns. Although early models were based on region-wide averages or nearest-monitor approaches, more current methods include land-use regression (LUR) (Hoek et al. 2008; Jerrett et al. 2007), the use of satellite and remote sensing data (Kloog et al. 2011), geostatistical methods such as kriging (Beelen et al. 2009), generalized additive models (Hart et al. 2009), or a combination of these techniques (Beckerman et al. 2013; Bergen et al. 2013; Mercer et al. 2011).

The Multi-Ethnic Study of Atherosclerosis and Air Pollution (MESA Air) is investigating the association between long-term air pollution exposure and measures of cardiovascular health (Kaufman et al. 2012). MESA Air is following a cohort of > 6,000 individuals in six metropolitan regions: Baltimore, Maryland; Chicago, Illinois; Los Angeles, California; New York, New York; St. Paul, Minnesota; and Winston-Salem, North Carolina. The primary exposures of interest in MESA Air are fine particulate matter (≤ 2.5 μm; PM_2.5_), nitrogen dioxide (NO_2_), oxides of nitrogen (NO_x_), and black carbon, as measured by light absorption coefficient (LAC). One goal of MESA Air is the development of advanced statistical methods that incorporate extensive supplemental monitoring to improve the prediction of intra-urban pollutant variability. MESA Air health effect analyses require spatiotemporal predictions of ambient outdoor concentrations for all four pollutants in all six metropolitan regions for times ranging from 1999 through 2012.

In general, exposure prediction models developed in one city do not transfer well to another city (Allen et al. 2011), so prediction models are often study- and city-specific (e.g., Franklin et al. 2012). A challenge for multi-city studies such as MESA Air that combine data from subcohorts and include several pollutant measures is generating predictions that are of comparable quality across pollutants and cities. Here we present a unified and flexible spatiotemporal modeling framework for the four MESA Air pollutants. We apply a standardized approach to model selection for all pollutants and regions, allowing the intrinsic flexibility of our modeling framework to account for differences in the way pollution processes behave in different regions.

## Methods

To predict outdoor concentration of pollutants at MESA Air participant residences, we fit a separate spatiotemporal exposure prediction model for each pollutant (PM_2.5_, NO_2_, NO_x_, and LAC) in each metropolitan region. Briefly, our model decomposes the space–time field of concentrations into spatially varying long-term (i.e., duration of study period) averages, spatially varying seasonal and long-term trends, and spatially correlated but temporally independent residuals, and accommodates data from the complex monitoring design described below. We modeled on a 2-week time scale because of the 2-week sampling of MESA Air supplementary monitoring instruments; this allows for flexible aggregation of predictions to time scales of interest for health effects analyses (e.g., 12 months before clinic visit).

*Monitoring data*. We used three sources of outdoor air monitoring data. We obtained hourly NO_2_ and NO_x_ and daily PM_2.5_ concentration measurements in each metropolitan region from 1 January 1999 through 31 March 2012 from the U.S. Environmental Protection Agency (EPA) Air Quality System (AQS) (http://www.epa.gov/ttn/airs/airsaqs/detaildata/downloadaqsdata.htm), including data from the Interagency Monitoring of Protected Visual Environments (IMPROVE) network (http://vista.cira.colostate.edu/IMPROVE/). No AQS data were used for black carbon because of differences in collection methods from the MESA Air LAC measurement methods described below. We aggregated hourly data into daily averages and subsequently averaged daily values to the 2-week scale. AQS monitors that had < 2 years of data or had irregular temporal coverage (e.g., operated only in the summer) were not used.

**Figure 1:**
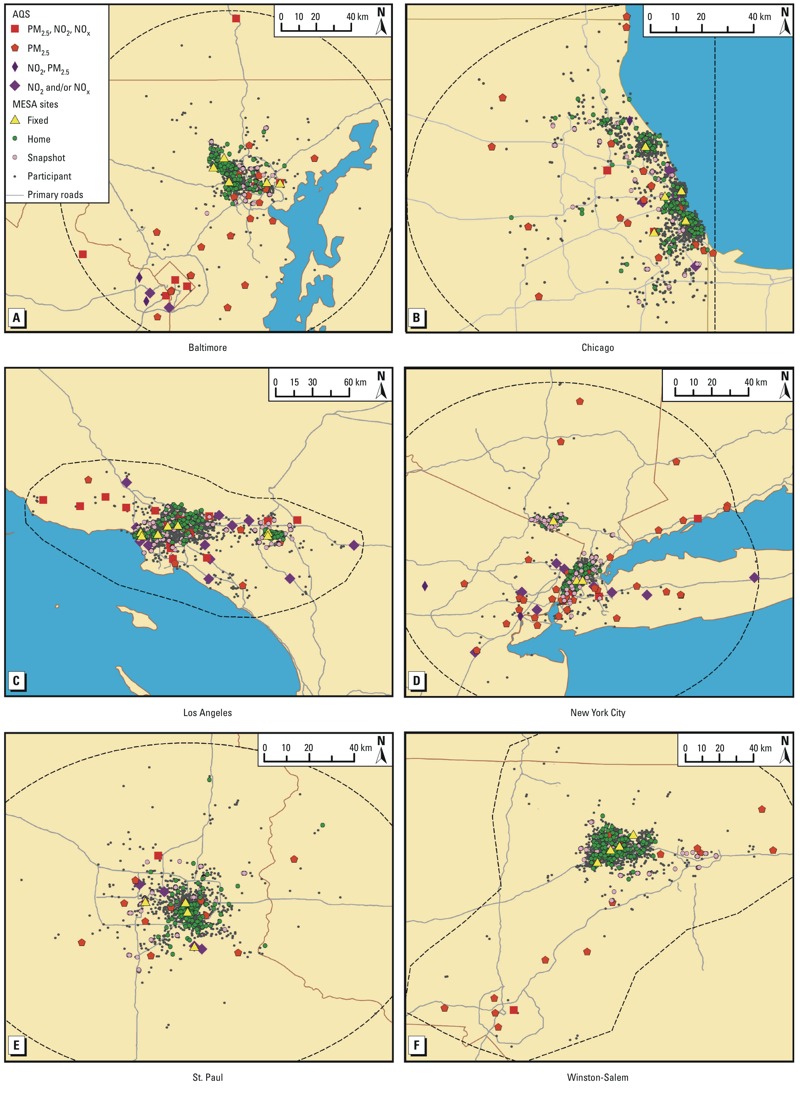
Maps of the modeling areas (denoted by dashed black line) in the six metropolitan regions, including monitor and subject locations. Abbreviations: Fixed, MESA Air fixed monitoring sites; Home, MESA Air home monitoring sites; Snapshot, MESA Air snapshot monitoring sites; Participant, MESA Air participant residence location (moved slightly to protect confidentiality).

In each metropolitan region, we defined the modeling area to be locations within approximately 75 km of each metropolitan center (Figure 1). AQS monitors within the modeling regions were considered for inclusion in the model, and predictions at participant residences were restricted to locations within these modeling regions. In New York, MESA Air participants were recruited from both New York City and Rockland County, so the modeling region included locations near both areas. In Winston-Salem, only one AQS monitoring location for NO_2_ and NO_x_ met inclusion criteria. To have a complete time series for the 14-year modeling period, an AQS monitor in Charlotte, North Carolina, was included for estimating time trends. In Chicago, the modeling region was further restricted to locations west of –87.5°W longitude because some covariates were unavailable east of that meridian. In Los Angeles, only locations south and/or west of the San Gabriel Mountains were included.

To better capture the within-city variability of pollutant concentrations, MESA Air conducted a supplementary monitoring campaign targeting the study cohort from July 2005 through August 2009. The MESA Air measurements were 2-week cumulative measurements that began and ended on Wednesdays. Measurements of NO_2_ and NO_x_ were made using Ogawa passive samplers, and PM_2.5_ mass was measured on Harvard Personal Environmental Monitor impactors using Teflon filters. LAC was computed from the Teflon filters via reflectance. A detailed description of the data collection and site selection procedures has been previously published (Cohen et al. 2009).

The MESA Air monitoring campaign included three types of monitoring sites: fixed, home, and snapshot. Fixed sites were operated for the duration of the 4-year MESA Air sampling period to provide long time series of measurements, with one fixed site collocated with an AQS monitor in each region. Samples of participant residences in each metropolitan region were selected for monitoring as home outdoor sites on a rotating basis, with most locations monitored one to three times in different seasons. Snapshot sites, which measured only NO_2_ and NO_x_, were located in clusters to capture gradients near sources (e.g., primary roadways) and monitored for three 2-week periods, one each in winter, summer, and either spring or fall.

In New York City, data from the New York City Community Air Survey (NYCCAS) were used to supplement the AQS and MESA Air data (Matte et al. 2013; NYC Department of Health 2014). The NYCCAS data consist of 2-week measurements of PM_2.5_, NO_2_, NO_x_, and LAC collected during December 2008–December 2010 in a manner consistent with MESA Air sampling protocols. Five NYCCAS reference sites (one in each borough) collected measurements throughout the sampling period, and 150 NYCCAS distributed sites were monitored once per season during this time. Because of the similarity in monitoring scheme, we treated NYCCAS reference sites in the same manner as MESA Air fixed sites, and NYCCAS distributed sites in the same manner as MESA Air home sites, in our models. The NYCCAS data and a small subset of the MESA Air 2-week data were centered on different weeks than most of the MESA Air measurements. To align these measurements with the rest of the MESA Air data, we treated these measurements as if they were made 1 week earlier or later, as appropriate.

Between 0.4% (LAC) and 1.6% (NO_2_) of the pollutant measurements were below the limit of detection (LOD) and were replaced with the value LOD/2. The number of each type of monitoring site by region and pollutant is provided in Table 1. The range of the number of PM_2.5_ observations at each monitoring site during the study period is provided in Table 2, along with summary statistics for the site means. Corresponding statistics for NO_2_, NO_x_, and LAC observations are provided in Supplemental Material, Tables S1–S3.

**Table 1:** Number of monitors by site type, region, and pollutant.

Site type	PM_2.5_	NO_2_	NO_x_	LAC
Baltimore, MD
AQS	29	11	8
MESA fixed	5	5	5	5
MESA home	86	87	87	86
MESA snapshot		104	104
Chicago, IL
AQS	20	7	6	
MESA fixed	6	6	6	6
MESA home	136	113	113	136
MESA snapshot		129	129
Los Angeles, CA
AQS	23	29	30
MESA fixed	7	7	7	7
MESA home	113	120	120	113
MESA snapshot		252	250
New York, NY
AQS	45	17	11
MESA fixed	3	3	3	3
MESA home	107	119	118	107
MESA snapshot		157	157
NYCCAS reference	5	5	5	5
NYCCAS distributed	150	150	150	150
St. Paul, MN
AQS	13	5	5
MESA fixed	3	4	4	3
MESA home	126	132	132	129
MESA snapshot		107	107
Winston-Salem, NC
AQS	16	2	2
MESA fixed	4	4	4	4
MESA home	114	117	117	114
MESA snapshot		121	121

**Table 2:** Summary of PM_2.5_ monitoring data.

Site type	No. of observations per site		Site means (μg/m^3^)		
	Minimum	Maximum	Minimum	Maximum	Mean ± SD
Baltimore, MD
AQS	64	345	10.9	16.9	13.4 ± 1.4
MESA fixed	18	92	12.1	15.4	13.7 ± 1.25
MESA home	1	3	7.3	22.7	14.3 ± 3.1
Chicago, IL
AQS	71	320	11.7	16.4	14.0 ± 1.3
MESA fixed	6	87	12.2	14.0	13.1 ± 0.75
MESA home	1	4	5.2	19.5	11.5 ± 3.2
Los Angeles, CA
AQS	82	345	10.7	22.8	16.2 ± 3.5
MESA fixed	76	85	13.7	19.3	16.2 ± 2.0
MESA home	1	2	0.7	42.6	16.9 ± 6.1
New York, NY
AQS	51	342	9.3	17.1	12.5 ± 1.8
MESA fixed	49	83	11.5	15.7	13.7 ± 2.1
MESA home	1	3	3.5	41.6	15.1 ± 4.9
NYCCAS reference	51	52	8.8	9.9	9.4 ± 0.42
NYCCAS distributed	6	8	6.8	19.8	11.0 ± 11.0
St. Paul, MN
AQS	55	305	7.9	11.6	10.0 ± 0.91
MESA fixed	81	89	9.6	10.5	10.0 ± 0.46
MESA home	1	5	5.0	27.4	10.3 ± 3.8
Winston-Salem, NC
AQS	86	346	10.3	15.9	13.4 ± 1.5
MESA fixed	80	93	13.0	13.8	13.4 ± 0.35
MESA home	1	4	9.0	22.8	14.3 ± 2.6


*Geographic covariates*. We compiled > 300 geographic covariates for use in the model (see Supplemental Material, Table S4). These covariates included proximity measures (distance to nearest major road, intersection, truck route, railway, railyard, coastline, airport, and port) and buffer measures (major road length, truck route length, land-use category, long-term vegetation index, population density, and emission sources). We included a long-term average of the dispersion model output from a modified implementation of the Caline3QHCR line-source model (Eckhoff and Braverman 1995). The Caline3QHCR model incorporates distance, traffic volume, meteorology, and diurnal traffic patterns in each region.

Geographic covariates with minimal variation or potentially highly influential values were excluded from the modeling process. Specifically, variables were removed if *a*) > 80% of monitoring sites had the same value, *b*) > 2% of observations were more than 5 SDs away from mean, *c*) the standard deviation of the distribution of values at participant residences was more than five times the standard deviation of the distribution of values at monitoring locations, or *d*) the maximum value was 10% among all monitoring sites (for land-use variables only). These filters were applied separately for each pollutant and region.

*Spatiotemporal model*. The monitoring data were highly unbalanced, with a small number of locations providing long time series of several years’ duration and a larger number of locations providing broader spatial coverage, but at a relatively small number of time points. A hierarchical spatiotemporal model had been previously developed to accommodate the unbalanced nature of the MESA Air data (Lindström et al. 2014; Sampson et al. 2011; Szpiro et al. 2010). This model can be written as

*C*(*s*,*t*) = *μ*(*s*,*t*) + *v*(*s*,*t*), [1]

where *C*(*s*,*t*) represents the log-transformed 2-week average pollutant concentration at location *s* and time *t*. The *μ*(*s*,*t*) term is the spatiotemporal mean surface, and the *v*(*s*,*t*) term represents spatiotemporal residual variation.

We break down the spatiotemporal mean into components


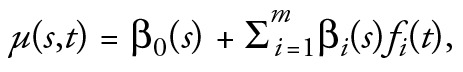
[2]

where β_0_(*s*) is the long-term mean at location *s*, *f_i_*(*t*) are smooth time trends, and β*_i_*(*s*) are spatially varying coefficients for the time trends.

The time trends are estimated from AQS and MESA Air fixed sites (and NYCCAS reference sites in New York) using a procedure developed by Fuentes et al. (2007) and previously described in detail by Sampson et al. (2011). In brief, we applied an expectation-maximization procedure to fill in missing values in the time series and derived the trends from a singular value decomposition. We smoothed the trends using splines, controlling the smoothness with the degrees of freedom (df) parameter. The model assumes the time trends account for enough of the temporal structure that the residuals are uncorrelated in time.

The long-term averages β_0_(*s*) and time trend coefficients β*_i_*(*s*) are modeled as spatial random fields with a spatial mean, distributed as

β*_i_ ~ N*[***X****_i_*(*s*)α*_i_,* Σ*_i_*(φ*_i_,* σ*_i_,* τ*_i_*)], *i* = 0, 1, …, *m*. [3]

Here, ***X****_i_*(*s*) are reduced-dimension summaries of the geographic covariates (described in detail below) at location *s*, and α*_i_* are vectors of coefficients to be estimated. The covariance structure for β*_i_*, denoted by Σ*_i_*, is either an independence model with variance τ*_i_* or a spatial smoothing model with exponential covariance function parameterized by range φ*_i_,* partial sill σ*_i_,* and nugget τ*_i_* (Cressie 1993).

The zero-mean spatiotemporal residual term *v*(*s*,*t*) in Equation 1 has a spatial correlation structure and is assumed independent at each time point. It includes a random effect for each time point to model short-term variations that affect an entire region, such as large-scale meteorological events.

*Partial least squares (PLS) scores*. Rather than include each of the hundreds of geographic covariates directly in the model or use variable selection methods, we reduced the dimensionality of the covariates using PLS. In a manner similar to principal components analysis (PCA), PLS computes linear combinations, called scores, of the columns of a data matrix. Unlike PCA, the PLS procedure constructs scores that maximize the covariance between the scores and an outcome rather than the variance between the scores. A technical explanation of the PLS algorithm is provided by Abdi (2010). Sampson et al. (2013) described the application of PLS for spatial models, and here we describe how we applied the method to spatiotemporal data.

PLS regression requires a single outcome value for each location. Because the MESA Air data are unbalanced time series, we first derived values that could be used as outcomes in PLS regression. For each AQS, fixed, and NYCCAS reference site *s*, we regressed the time series of observations *C*(*s*,*t*) on the smoothed time trends using ordinary least squares regression with mean function E[*C*(*s*,*t*)] = γ^s^_0_ *f*_0_(*t*) + … + γ^s^_m_ *f*_m_(*t*) to get estimates (^^^γ^s^_0_,…, ^^^γ^s^_m_) for each location. For each time trend, PLS regression was performed separately with the ^^^γ^s^_i_ as the outcomes and the matrix of geographic covariates as the predictors. This gave a set of PLS scores for each location that was different for each time trend. PLS scores at home and snapshot monitoring sites were predicted using the geographic variables at those locations and the score definitions defined from the regression at fixed sites. PLS regression was performed using the pls package (Mevik et al. 2011), in R (R Core Team; http:r-project.org). Scores were included in the model as the ***X****_i_*(*s*) in Equation 3.

*Parameter estimation and model selection*. Once the PLS scores ***X****_i_*(*s*) and time trends *f_i_*(*t*) were computed, the remaining parameters were calculated via maximum likelihood using the SpatioTemporal package, version 1.1.7 (Lindström et al. 2012), in R. We varied several model parameters and used cross-validation to find the best-fitting model in each metropolitan region, as described below. We considered different values for the number of time trends (either 1 or 2), the df for smoothing time trends (either 4 or 8 per year), the number of PLS scores per time trend (2 or 3), and the covariance structure of the β*_i_* fields (spatial smoothing or no spatial smoothing).

*Cross-validation procedure*. The primary interest of MESA Air is in long-term average exposures, so we assessed model performance using cross-validation of long-term averages (LTAs). Because the highly unbalanced structure of the monitoring data means that LTAs at home sites are computed from a handful of observations of a few weeks’ duration, whereas LTAs at fixed sites are computed from long time series, we performed cross-validation separately for each site type. For home sites and NYCCAS distributed sites, we used 10-fold cross-validation, which leaves out one-tenth of the data in turn. For AQS, fixed, and NYCCAS references sites, we used leave-one-out cross-validation because the total number of monitors was relatively small. For snapshot sites, we used 10-fold cross-validation, with monitors in the same cluster left out together. For all three schemes, the covariance parameters (but not the time trends or PLS scores) were re-estimated using all but the left-out sites. Pollutant concentrations at the left-out sites were predicted using the parameters estimated from the remaining data.

We assessed cross-validation performance using two measures: root mean-squared error (RMSE) and cross-validation *R*^2^ (denoted by *R*^2^*_CV_*). Letting *y_j_* denote the mean observed value and ^^^*y_j_* the mean of the predicted values for the observed time points at monitoring site *j*, RMSE and *R*^2^*_CV_* were computed on the original scale of the data according to the formulas


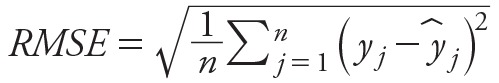
[4]

and

*R*^2^*_CV_* = max(0,1 – *RMSE*^2^/*MSE_obs_*), [5]

where *MSE_obs_* = 1/*n* Σ*^n^_j_*_= 1_ (*y_j_* – ^–^*y*)^2^ is the mean-squared error of the observed values. *R*^2^*_CV_* provides a measure of fit to the 1-1 line, in contrast to the typical regression-based *R*^2^ (*R*^2^*_CVreg_*), which measures fit to the regression line and is computed as the square of the correlation coefficient between the cross-validation predictions and the observed values. *R*^2^*_CV_* reflects the contrast of interest because our goal is accurate prediction at unmeasured locations, and it is typically lower than *R*^2^*_CVreg_*. Although *R*^2^*_CV_* was the primary metric for our model evaluation, we present *R*^2^*_CVreg_* for comparison with published results from other authors. Because we are most interested in spatial contrasts between individual exposures within each region, we prioritized home-site over fixed-site *R*^2^*_CV_* and RMSE in the model selection process.

In the context of the hierarchical model (Equation 1), it is challenging to separate the spatial and temporal contributions to *R*^2^*_CV_* for cross-validation of temporally sparse data sets such as the home sites and NYCCAS distributed sites. Lindström et al. (2014) proposed three temporally adjusted adaptations of *R*^2^*_CV_* that use data from the AQS and fixed sites as the reference MSE instead of *MSE_obs_* in Equation 5 in order to focus on spatial prediction accuracy. *R*^2^*_Avg_* uses the average values at AQS and fixed sites within that region. *R*^2^*_Close_* uses the closest (in absolute distance) AQS or fixed site. *R*^2^*_Smooth_* uses the smoothed time trend at the closest AQS or fixed site.

*Prediction at participant locations*. Using the best models from each metropolitan region, predictions of pollutant log-concentrations at participant residences were made on a 2-week scale from January 1999 through March 2012. We back-transformed these predictions using exponentiation to return them to the original scale of concentration measurements and computed averages of 2-week predictions over the study period.

## Results

**Figure 2:**
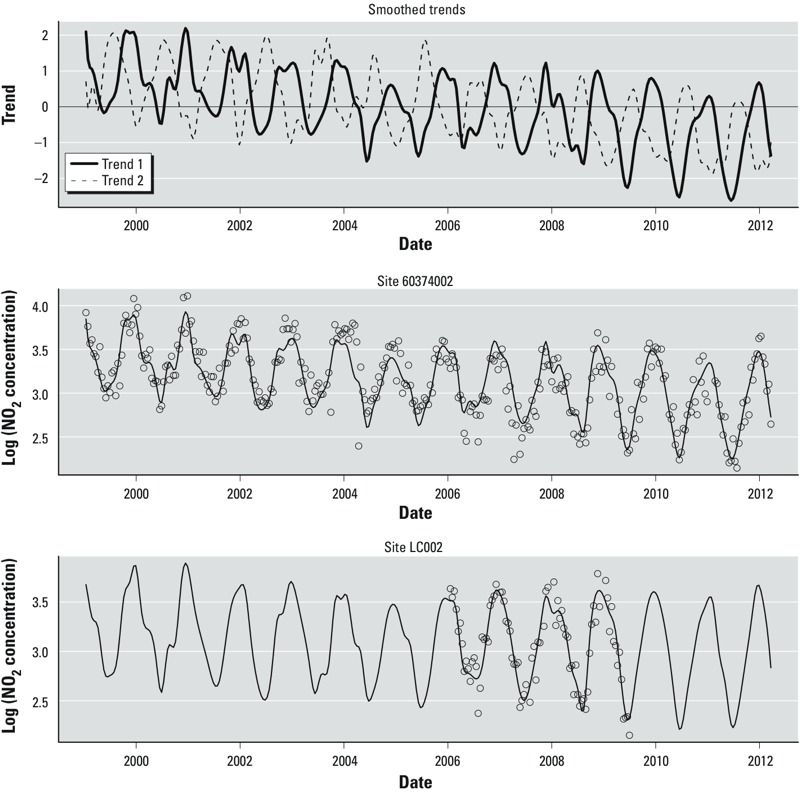
Time trends for the NO_2_ model in Los Angeles. The top panel shows the smoothed time trends calculated from AQS and fixed sites. The middle and bottom panels show the observed data and fitted trends at an AQS site and fixed site, respectively.

*Model structure*. Table 3 provides an overview of the model structure selected for each metropolitan region and pollutant. Most models have only one time trend, although all the New York models have two. The two smoothed trends for the NO_2_ model in Los Angeles are shown in Figure 2. Figure 2 also includes plots of the fitted trends for a selected AQS site and fixed site.

**Table 3:** Model structure for the best model (selected by cross-validation) for each pollutant and metropolitan region.

Model	No. of time trends^*a*^	No. of PLS scores^*b*^	df/year in time trend^*c*^	Spatial smoothing^*d*^	
				Long-term average (β_0_)	Time trend coefficients (β_i_)
Baltimore, MD
PM_2.5_	1	3	4	Yes	No
NO_2_	1	2	8	Yes	No
NO_x_	1	2	8	Yes	Yes
LAC	1	3	8	No	No
Chicago, IL
PM_2.5_	1	3	8	Yes	No
NO_2_	2	2	4	Yes	Yes
NO_x_	2	2	8	Yes	No
LAC	1	2	8	Yes	Yes
Los Angeles, CA
PM_2.5_	2	3	8	Yes	No
NO_2_	2	3	8	Yes	Yes
NO_x_	1	3	4	Yes	Yes
LAC	1	2	4	Yes	No
New York, NY
PM_2.5_	2	3	8	No	No
NO_2_	2	3	4	Yes	Yes
NO_x_	2	2	8	No	No
LAC	2	2	4	Yes	No
St. Paul, MN
PM_2.5_	1	3	4	Yes	No
NO_2_	1	3	4	Yes	No
NO_x_	1	3	4	Yes	No
LAC	1	2	8	Yes	No
Winston‑Salem, NC
PM_2.5_	2	2	4	No	No
NO_2_	1	3	8	Yes	Yes
NO_x_	1	2	8	Yes	Yes
LAC	1	2	8	Yes	No
^***a***^Selected from either 1 or 2 time trends. ^***b***^Selected from either 2 or 3 PLS scores; scores were covariates in the mean component of the long-term average (β_0_) and time trend (β_i_) fields [denoted by *X*_*i*_(*s*) in Equation 3]. ^***c***^Selected from either 4 or 8 degrees of freedom (df) per year; controls smoothness of estimated time trends. ^***d***^Yes, exponential covariance structure. No, independent covariance structure.					

For PM_2.5_, there was noticeable heterogeneity of the best models across metropolitan regions (Table 3). New York and Winston-Salem had no spatial smoothing in the long-term PM_2.5_ average [β_0_(s)], and no model had spatial smoothing in the PM_2.5_ time trend coefficients [β*_i_*(*s*)]. Half of the regions had two time trends, whereas the other half had only a single time trend for PM_2.5_. For NO_2_, all of the models had spatial smoothing for the long-term average, and the same was true for NO_x_ except in New York.

The relative contribution of geographic covariates to the PLS scores varied by region and pollutant. In the Supplemental Material, Figure S1 shows the correlations between covariates and PLS scores for the NO_2_ model in Chicago, which are representative of the general patterns in the other regions (data not shown). Overall, the distance-to-feature covariates and vegetation measures [Normalized Difference Vegetation Index (NDVI) and low development, open development, forest, and wetland land use] tended to have the opposite correlation from emissions and traffic measures (A1, A2/A3, and truck route lengths and intersection counts) within buffers.

**Figure 3:**
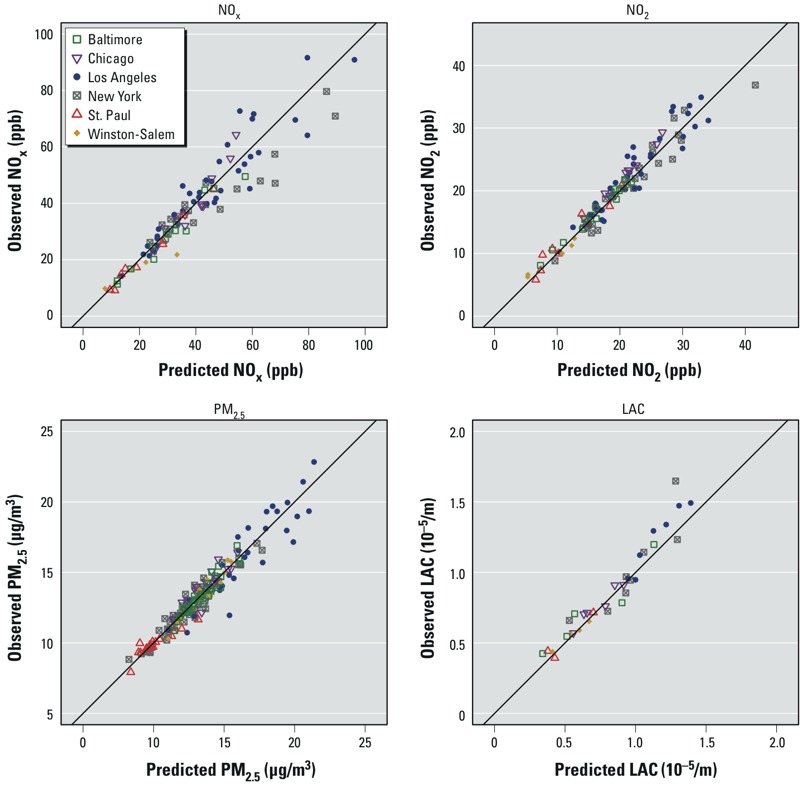
Long-term averages of cross-validated predictions and observations for AQS and fixed monitoring locations for each pollutant.

*Model results*. Table 4 shows the cross-validation metrics for all models, broken down by pollutant and region. These metrics assess how well the site means are modeled, incorporating both the spatial and temporal components of the predictions. Scatter plots of predictions and observed values are provided in Figure 3 for AQS and fixed sites and in Supplemental Material, Figure S2, for home sites. Metrics for the snapshot sites (for NO_2_ and NO_x_) and for AQS and fixed sites (all four pollutants) on the 2-week scale are reported in the Supplemental Material, Tables S5 and S6.

**Table 4:** Cross-validation measures of predictive accuracy for site means at monitoring locations.

Region	AQS and MESA fixed sites			MESA home sites		
	RMSE^*a*^	*R*^2^_*CV*_	*R*^2^_*CVreg*_	RMSE^*a*^	*R*^2^_*CV*_	*R*^2^_*CVreg*_
PM_2.5_
Baltimore	0.42	0.90	0.90	1.24	0.84	0.86
Chicago	0.59	0.78	0.82	1.43	0.80	0.80
Los Angeles	1.28	0.83	0.84	2.92	0.77	0.78
New York^*b*^	0.59	0.91	0.91	2.80	0.54	0.56
St. Paul	0.60	0.45	0.84	1.78	0.78	0.79
Winston-Salem	0.44	0.89	0.90	1.00	0.85	0.86
NO_2_
Baltimore	0.76	0.96	0.97	1.47	0.90	0.90
Chicago	1.51	0.87	0.97	3.31	0.45	0.48
Los Angeles	2.23	0.88	0.89	3.13	0.77	0.78
New York^*b*^	1.86	0.92	0.93	3.82	0.78	0.78
St. Paul	1.27	0.93	0.94	1.24	0.87	0.87
Winston-Salem	0.95	0.85	0.98	1.41	0.74	0.75
NO_x_
Baltimore	3.32	0.92	0.96	3.98	0.92	0.92
Chicago	3.88	0.87	0.91	6.08	0.59	0.59
Los Angeles	6.74	0.87	0.87	5.69	0.91	0.92
New York^*b*^	8.85	0.61	0.89	16.66	0.50	0.50
St. Paul	1.69	0.98	0.98	3.58	0.83	0.84
Winston-Salem	5.46	0.00	0.94	3.74	0.60	0.63
LAC
Baltimore	0.096	0.87	0.91	0.127	0.78	0.79
Chicago	0.045	0.86	0.92	0.108	0.61	0.62
Los Angeles	0.114	0.70	0.93	0.266	0.69	0.71
New York^*b*^	0.147	0.75	0.79	0.329	0.51	0.52
St. Paul	0.043	0.91	0.92	0.074	0.69	0.69
Winston-Salem	0.020	0.94	0.99	0.088	0.64	0.65
^***a***^Units for RMSE are μg/m^3^ (PM_2.5_), ppb (NO_2_ and NO_x_), and 10^–5^/m (LAC). ^***b***^New York models include NYCCAS reference sites with AQS and fixed sites, and NYCCAS distributed sites with home sites.						

Predictive accuracy was generally good (*R*^2^*_CV_* > 0.6) to excellent (*R*^2^*_CV_* > 0.8) in almost all regions for each pollutant. NO_x_ models in Baltimore and Los Angeles had the best performance at MESA home sites (*R*^2^*_CV_* of 0.92 and 0.91, respectively) (Table 4). The lowest *R*^2^*_CV_* for home sites was in the Chicago NO_2_ model (0.45), but its RMSE (3.31 ppb) was comparable with those in New York and Los Angeles (3.82 and 3.13 ppb, respectively). At AQS and fixed sites, *R*^2^*_CV_* was very good (0.70 for LAC in Los Angeles) to excellent (0.98 for NO_x_ in St. Paul), with two notable exceptions: Winston-Salem NO_x_ (0.00) and St. Paul PM_2.5_ (0.45). However, in both cases the RMSE was comparable with the corresponding RMSE for models in other regions. The small range of observed data (9.8–22.4 ppb) (see Supplemental Material, Table S2) and the small number of monitors in Winston-Salem (Table 1) explain the low *R*^2^*_CV_* for NO_x_ in that city.

Table 5 provides three versions of temporally adjusted *R*^2^*_CV_* at home sites (*R*^2^*_Avg_*, *R*^2^*_Close_*, and *R*^2^*_Smooth_*). For NO_2_ and NO_x_, these temporally adjusted *R*^2^*_CV_* are fairly similar to the unadjusted *R*^2^*_CV_* reported in Table 4, suggesting that we are predicting spatial differences well. For PM_2.5_, however, the temporally adjusted *R*^2^*_CV_* are consistently lower than the unadjusted *R*^2^*_CV_*.

**Table 5:** Temporally adjusted cross-validation measures of predictive accuracy for home site means.

Region	*R*^2^_*Avg*_	*R*^2^_*Close*_	*R*^2^_*Smooth*_
PM_2.5_
Baltimore	0.45	0.52	0.72
Chicago	0.23	0.33	0.64
Los Angeles	0.40	0.23	0.43
New York^*a*^	0.48	0.36	0.38
St. Paul	0.23	0.29	0.62
Winston-Salem	0.29	0.60	0.77
NO_2_
Baltimore	0.92	0.79	0.74
Chicago	0.73	0.64	0.78
Los Angeles	0.63	0.66	0.66
New York^*a*^	0.89	0.78	0.64
St. Paul	0.77	0.89	0.90
Winston-Salem	0.73	0.79	0.81
NO_x_
Baltimore	0.86	0.70	0.65
Chicago	0.76	0.73	0.69
Los Angeles	0.81	0.85	0.88
New York^*a*^	0.72	0.64	0.52
St. Paul	0.79	0.88	0.85
Winston-Salem	0.43	0.62	0.64
LAC
Baltimore	0.78	0.67	0.32
Chicago	0.56	0.45	0.36
Los Angeles	0.28	0.34	0.48
New York^*a*^	0.59	0.65	0.53
St. Paul	0.67	0.80	0.84
Winston-Salem	0.37	0.56	0.59
General formula for *R*^2^ measures is *R*^2^ = *max *(0,1 – *RMSE*^*2*^*/MSE*_*obs*_). *R*^2^_*Avg*_ uses the mean-squared error of the average observed values at AQS and fixed sites within the region as *MSE*_*obs*_. *R*^2^_*Close*_ uses the mean-squared error of the observed values at the closest AQS or Fixed site as *MSE*_*obs*_. *R*^2^_*Smooth*_ uses the mean-squared error of the values of the smoothed time trend at the nearest AQS or Fixed site as *MSE*_*obs*_. ^***a***^Includes NYCCAS distributed sites.			

**Figure 4:**
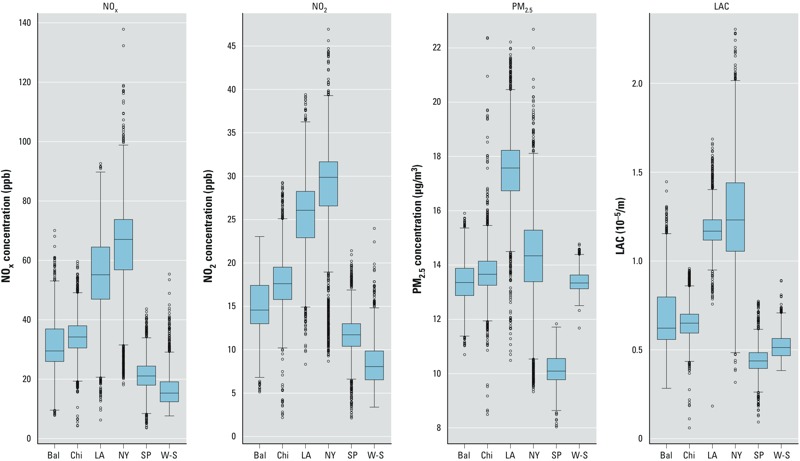
Pollutant- and region-specific box plots of long-term averages of predictions from 1999 through early 2012 at participant residence locations. Metropolitan region abbreviations: Bal, Baltimore; Chi, Chicago; LA, Los Angeles; NY, New York; SP, St. Paul; W-S, Winston-Salem. Boxes extend from the 25th to the 75th percentile, horizontal bars represent the median, whiskers extend 1.5 times the length of the interquartile range above and below the 75th and 25th percentiles, respectively, and outliers are presented as points.

Box plots of the long-term averages of predictions at participant residences are provided in Figure 4. On average, predicted concentrations tended to be higher in New York and Los Angeles, consistent with the higher observed monitoring values in those regions. Variability in predictions is also greatest in these two cities, especially in the tails of the distributions.

Supplemental Material, Table S7, provides performance metrics for the New York models when the NYCCAS data were excluded from the modeling process. Without the NYCCAS data, *R*^2^*_CV_* was noticeably lower for PM_2.5_ (0.79 vs. 0.91 at AQS and fixed sites, and 0.36 vs. 0.54 at home sites) and LAC (0.55 vs. 0.75 at AQS and fixed sites, and 0.43 vs. 0.51 at home sites).

## Discussion

We present here a complex and successful approach to predicting long-term air pollution concentrations for application in a cohort study. Although this approach was tailored to this particular well-characterized cohort study—taking advantage of cohort-specific monitoring, for example—the success of the approach demonstrates modeling improvements that can be adopted for application in future population-based research on spatially varying pollutants. We believe that this approach to capturing variation in within-region pollution highlights advances that should be adopted in the next generation of air pollution cohort studies, both for understanding contrasts at relatively low concentrations in the United States and at the higher concentrations experienced globally.

We describe a unified framework for implementing exposure prediction models of four air pollutants in six metropolitan regions that easily incorporates spatially and temporally unbalanced monitoring data. The application of a consistent modeling framework to all regions and pollutants is important for studies such as MESA Air that use exposure estimates from multiple subcohorts together in health analyses. Although we applied the same approach in all regions, we varied the model structure to best fit the data for each region and pollutant. This unified modeling approach was shown to have very good model performance (*R*^2^*_CV_* > 0.70) for almost all of the pollutants and regions. The architecture for this modeling approach is publicly available through the SpatioTemporal R package (Lindström et al. 2012).

As a result of the success of our spatiotemporal modeling approaches, we are confident in using these approaches to model outdoor pollutant concentrations in epidemiological analyses in this cohort and in other populations residing in these same communities. We have also found that implementation of portions of this approach, such as PLS regression of a large set of geographic covariates combined with spatial smoothing via universal kriging, can be used with good success in other regions to predict pollutant concentrations without the same level of small-area monitoring (Sampson et al. 2013). The NYCCAS data increased the spatial density of the monitoring data in New York, which was likely one reason for the improved model performance when the data were included. For LAC, the NYCCAS data provided particular benefit because they allowed the model to be extended through 2010, which would not have been possible with only the MESA Air data.

A majority of models included spatial smoothing in the long-term average. This suggests that although PLS scores derived from geographic covariates can predict much of the spatial variation in the data, benefit is gained from borrowing strength across observations nearby in space.

Differences in the underlying pollutant variability likely caused some of the differences seen in temporally adjusted *R*^2^*_CV_*. PM_2.5_ tended to exhibit less small-scale spatial variation and greater temporal variability, leading to temporally adjusted *R*^2^*_CV_* that are noticeably lower than the unadjusted measures. The NO_2_ and NO_x_ data tended to exhibit greater spatial variability, and the similarity of the unadjusted and temporally adjusted *R*^2^*_CV_* values suggests that the unadjusted *R*^2^*_CV_* are not overly inflated by well-predicted temporal variation.

The modeling approach presented here does have several limitations. First, we used geographic covariates that were constant in time [although the modeling framework readily extends to spatiotemporal covariates (Lindström et al. 2014)]. Changes in these variables likely occurred during the study decade, but we nonetheless believe that the time-constant geographic variables still provided a useful means to predict long-term pollutant concentrations. Second, the calculation of PLS scores was limited to AQS and fixed sites because they had long time series. For LAC in particular, this means that the scores were based on a very small number of locations because the LAC model relied only on MESA Air data (plus NYCCAS data for New York). Third, the cross-validation model selection procedure conditioned on the time trends and PLS scores. Overfitting may have occurred in the cross-validation of the AQS and fixed sites, because the left-out observations were used to estimate the time trends and PLS scores. However, because the home sites were not used in estimating time trend or in defining the PLS scores, any overfitting was restricted to the AQS and fixed-site cross-validation. This provides further motivation for prioritization of cross-validation metrics from home sites when selecting the best models.

## Conclusions

Our unified spatiotemporal modeling method successfully characterized outdoor concentrations of multiple air pollutants at the homes of cohort members in multiple metropolitan regions. This flexible and powerful modeling approach can incorporate an unbalanced monitoring data structure, leveraging data from supplemental monitoring campaigns that increase the spatial coverage of monitoring data. The method was easily transferred between regions and pollutants, allowing for straightforward comparison between model fits across regions. Although aspects of our techniques are particularly tailored to the unique data and resources of MESA Air, lessons learned here can be applied to understand the spatial and temporal variation of pollutants in future cohort studies. Advances in fine-scale modeling resolved in both space and time are important for the next generation of cohort studies assessing health effects of environmental agents.

## Supplemental Material

(1.7 MB) PDF

## References

[r1] Abdi H 2010 Partial least squares regression and projection on latent structure regression (PLS Regression). Wiley Interdiscip Rev Comput Stat 2 97 106; 10.1002/wics.51

[r2] Allen RW Amram O Wheeler AJ Brauer M 2011 The transferability of NO and NO_2_ land use regression models between cities and pollutants. Atmos Environ 45 369 378; 10.1016/j.atmosenv.2010.10.002

[r3] Beckerman BS Jerrett M Serre M Martin RV Lee SJ van Donkelaar A 2013 A hybrid approach to estimating national scale spatiotemporal variability of PM_2.5_ in the contiguous United States. Environ Sci Technol 47 7233 7241; 10.1021/es400039u 23701364 PMC3976544

[r4] Beelen R Hoek G Pebesma E Vienneau D de Hoogh K Briggs DJ 2009 Mapping of background air pollution at a fine spatial scale across the European Union. Sci Total Environ 407 1852 1867; 10.1016/j.scitotenv.2008.11.048 19152957

[r5] Bergen S Sheppard L Sampson PD Kim SY Richards M Vedal S 2013 A national prediction model for PM_2.5_ component exposures and measurement error-corrected health effect inference. Environ Health Perspect 121 1017 1025; 10.1289/ehp.1206010 23757600 PMC3764074

[r6] Brook RD Rajagopalan S Pope CA III Brook JR Bhatnagar A Diez-Roux AV 2010 Particulate matter air pollution and cardiovascular disease: an update to the scientific statement from the American Heart Association. Circulation 121 2331 2378; 10.1161/CIR.0b013e3181dbece1 20458016

[r7] Cohen MA, Adar SD, Allen RW, Avol E, Curl CL, Gould T (2009). Approach to estimating participant pollutant exposures in the Multi-Ethnic Study of Atherosclerosis and Air Pollution (MESA Air).. Environ Sci Technol.

[r8] Cressie NAC. (1993). Statistics for Spatial Data, Revised Edition..

[r9] Eckhoff P, Braverman T. (1995). Addendum to the User’s Guide to CAL3QHC Version 2.0 (CAL3QHCR user’s guide).. http://www.epa.gov/scram001/userg/regmod/cal3qhcrug.pdf.

[r10] Franklin M Vora H Avol E McConnell R Lurmann F Liu F 2012 Predictors of intra-community variation in air quality. J Expo Sci Environ Epidemiol 22 135 147; 10.1038/jes.2011.45 22252279 PMC4391642

[r11] Fuentes M, Guttorp P, Sampson PD. (2007). Using transforms to analyze space-time processes.

[r12] Hart JE Yanosky JD Puett RC Ryan L Dockery DW Smith TJ 2009 Spatial modeling of PM_10_ and NO_2_ in the continental United States, 1985–2000. Environ Health Perspect 117 1690 1696; 10.1289/ehp.0900840 20049118 PMC2801201

[r13] Hoek G Beelen R de Hoogh K Vienneau D Gulliver J Fischer P 2008 A review of land-use regression models to assess spatial variation of outdoor air pollution. Atmos Environ 42 7561 7578; 10.1016/j.atmosenv.2008.05.057

[r14] Jerrett M Arain M Kanaroglou P Beckerman B Crouse D Gilbert NL 2007 Modeling the intraurban variability of ambient traffic pollution in Toronto, Canada. J Toxicol Environ Health A 70 200 212; 10.1080/15287390600883018 17365582

[r15] Kaufman JD Adar SD Allen RW Barr RG Budoff MJ Burke GL 2012 Prospective study of particulate air pollution exposures, subclinical atherosclerosis, and clinical cardiovascular disease: the Multi-Ethnic Study of Atherosclerosis and Air Pollution (MESA Air). Am J Epidemiol 176 825 837; 10.1093/aje/kws169 23043127 PMC3571256

[r16] Kloog I Koutrakis P Coull BA Lee HJ Schwartz J 2011 Assessing temporally and spatially resolved PM_2.5_ exposures for epidemiological studies using satellite aerosol optical depth measurements. Atmos Environ 45 6267 6275; 10.1016/j.atmosenv.2011.08.066

[r17] Lindström J, Szpiro AA, Sampson PD, Bergen S, Oron AP. (2012). SpatioTemporal: Spatio-Temporal Model Estimation. R Package Version 1.1.7.. http://cran.r-project.org/web/packages/SpatioTemporal/index.html.

[r18] Lindström J Szpiro AA Sampson PD Oron AP Richards M Larson TV 2014 A flexible spatio-temporal model for air pollution with spatio-temporal covariates. Environ Ecol Stat 21 411 433; 10.1007/s10651-013-0261-4 25264424 PMC4174563

[r19] Matte TD Ross Z Kheirbek I Eisl H Johnson S Gorczynski JE 2013 Monitoring intraurban spatial patterns of multiple combustion air pollutants in New York City: design and implementation. J Expo Sci Environ Epidemiol 23 223 231; 10.1038/jes.2012.126 23321861

[r20] Mercer LD Szpiro AA Sheppard L Lindström J Adar SD Allen RW 2011 Comparing universal kriging and land-use regression for predicting concentrations of gaseous oxides of nitrogen (NO_x_) for the Multi-Ethnic Study of Atherosclerosis and Air Pollution (MESA Air). Atmos Environ 45 4412 4420; 10.1016/j.atmosenv.2011.05.043 PMC3146303 21808599

[r21] Mevik BH, Wehrens R, Liland KH. (2011). PLS: Partial Least Squares and Principal Component regression. R Package Version 2.3-0.. http://cran.r-project.org/web/packages/pls/index.html.

[r22] Miller KA, Siscovick DS, Sheppard L, Shepherd K, Sullivan JH, Anderson GL (2007). Long-term exposure to air pollution and incidence of cardiovascular events in women.. N Engl J Med.

[r23] NYC Department of Health. (2014). NYC Community Air Survey.. http://www.nyc.gov/health/nyccas.

[r24] Pope CA III Dockery DW 2006 Health effects of fine particulate air pollution: lines that connect. J Air Waste Manag Assoc 56 709 742; 10.1080/10473289.2006.10464485 16805397

[r25] Sampson PD Richards M Szpiro AA Bergen S Sheppard L Larson TV 2013 A regionalized national universal kriging model using partial least squares regression for estimating annual PM_2.5_ concentrations in epidemiology. Atmos Environ 75 383 392; 10.1016/j.atmosenv.2013.04.015 PMC3763950 24015108

[r26] Sampson PD Szpiro AA Sheppard L Lindström J Kaufman JD 2011 Pragmatic estimation of a spatio-temporal air quality model with irregular monitoring data. Atmos Environ 45 6593 6606; 10.1016/j.atmosenv.2011.04.073

[r27] Szpiro AA Sampson PD Sheppard L Lumley T Adar SD Kaufman JD 2010 Predicting intra-urban variation in air pollution concentrations with complex spatio-temporal dependencies. Environmetrics 21 606 631; 10.1002/env.1014 24860253 PMC4029437

