# Supplementary material for: A Unified Spatiotemporal Modeling Approach for Predicting Concentrations of Multiple Air Pollutants in the Multi-Ethnic Study of Atherosclerosis and Air Pollution
Source: Environ Health Perspect. 2014 Nov 14;123(4):301–9. doi: 10.1289/ehp.1408145 (PMC4384200; doi:10.1289/ehp.1408145)
Supplement: (1.7 MB) PDF [file ehp.1408145.s001.508.pdf]

*Environmental Health Perspectives* doi:10.1289/ehp.1408145

# Supplemental Material

# A Unified Spatiotemporal Modeling Approach for Predicting Concentrations of Multiple Air Pollutants in the Multi-Ethnic Study of Atherosclerosis and Air Pollution

Joshua P. Keller, Casey Olives, Sun-Young Kim, Lianne Sheppard, Paul D. Sampson, Adam A. Szpiro, Assaf P. Oron, Johan Lindström, Sverre Vedal, and Joel D. Kaufman

**Table S1.** Summary of $NO_2$ monitoring data.

| Region and site type | No. of observations per site Min, max | Site means (ppb) Min, max | Site means (ppb) Mean ± SD |
|---|---|---|---|
| **Baltimore, MD** | | | |
| AQS | 59, 343 | 11.7, 21.8 | 17.9 ± 3.0 |
| MESA Fixed | 27, 98 | 8.1, 20.4 | 13.5 ± 4.7 |
| MESA Home | 1, 3 | 3.0, 29.2 | 11.0 ± 4.6 |
| MESA Snapshot | 1, 3 | 6.5, 24.4 | 16.0 ± 4.0 |
| **Chicago, IL** | | | |
| AQS | 77, 339 | 15.7, 29.3 | 23.1 ± 4.7 |
| MESA Fixed | 8, 96 | 14.2, 23.3 | 18.8 ± 3.0 |
| MESA Home | 1, 5 | 0.5, 30.7 | 15.6 ± 4.5 |
| MESA Snapshot | 1, 3 | 7.4, 29.8 | 17.4 ± 4.6 |
| **Los Angeles, CA** | | | |
| AQS | 84, 342 | 10.2, 34.9 | 22.9 ± 6.8 |
| MESA Fixed | 81, 90 | 16.9, 32.3 | 22.4 ± 6.2 |
| MESA Home | 1, 2 | 8.7, 39.0 | 21.6 ± 6.6 |
| MESA Snapshot | 1, 3 | 11.9, 42.8 | 28.0 ± 6.3 |
| **New York, NY** | | | |
| AQS | 75, 343 | 8.8, 36.9 | 22.4 ± 7.2 |
| MESA Fixed | 63, 93 | 13.7, 32.8 | 23.9 ± 9.6 |
| MESA Home | 1, 3 | 8.2, 47.9 | 25.2 ± 8.1 |
| MESA Snapshot | 1, 3 | 6.8, 46.3 | 23.7 ± 10.0 |
| NYCCAS Reference | 51, 52 | 14.1, 21.8 | 19.2 ± 3.1 |
| NYCCAS Distributed | 7, 8 | 12.0, 56.1 | 26.2 ± 8.3 |
| **St. Paul, MN** | | | |
| AQS | 99, 331 | 7.3, 20.8 | 13.0 ± 5.5 |
| MESA Fixed | 64, 97 | 5.8, 17.5 | 12.0 ± 5.2 |
| MESA Home | 1, 5 | 1.3, 20.9 | 10.2 ± 3.4 |
| MESA Snapshot | 1, 3 | 2.6, 22.0 | 10.9 ± 4.1 |
| **Winston-Salem, NC** | | | |
| AQS | 306, 313 | 12.4, 13.8 | 13.1 ± 1.0 |
| MESA Fixed | 79, 100 | 6.3, 11.3 | 8.5 ± 2.5 |
| MESA Home | 1, 5 | 1.2, 17.2 | 6.5 ± 2.8 |
| MESA Snapshot | 1, 3 | 2.8, 27.5 | 9.4 ± 3.8 |

**Table S2.** Summary of $NO_X$ monitoring data.

| Region and site type | No. of observations per site<br>Min, max | Site means (ppb)<br>Min, max | Site means (ppb)<br>Mean ± SD |
|---|---|---|---|
| **Baltimore, MD** | | | |
| AQS | 130, 343 | 11.3, 45.3 | 29.9 ± 11.5 |
| MESA Fixed | 27, 98 | 12.5, 49.5 | 27.2 ± 14.4 |
| MESA Home | 1, 3 | 4.7, 99.6 | 20.4 ± 14.4 |
| MESA Snapshot | 1, 3 | 9.9, 60.5 | 35.3 ± 11.4 |
| **Chicago, IL** | | | |
| AQS | 77, 339 | 32.1, 64.3 | 46.5 ± 12.1 |
| MESA Fixed | 8, 96 | 23.3, 39.7 | 33.0 ± 5.5 |
| MESA Home | 1, 5 | 9.4, 72.2 | 27.6 ± 9.5 |
| MESA Snapshot | 1, 3 | 17.4, 69.4 | 34.4 ± 10.6 |
| **Los Angeles, CA** | | | |
| AQS | 88, 342 | 14.2, 91.6 | 45.4 ± 19.8 |
| MESA Fixed | 81, 90 | 36.0, 71.7 | 50.0 ± 13.4 |
| MESA Home | 1, 2 | 14.1, 107 | 44.7 ± 19.5 |
| MESA Snapshot | 1, 3 | 16.7, 141 | 66.0 ± 25.3 |
| **New York, NY** | | | |
| AQS | 73, 330 | 23.1, 70.9 | 42.4 ± 13.3 |
| MESA Fixed | 62, 92 | 29.0, 79.6 | 52.2 ± 25.6 |
| MESA Home | 1, 3 | 11.9, 180 | 49.4 ± 23.3 |
| MESA Snapshot | 1, 3 | 12.6, 155 | 60.8 ± 34.8 |
| NYCCAS Reference | 51, 52 | 26.1, 39.5 | 34.1 ± 5.2 |
| NYCCAS Distributed | 7, 8 | 20.6, 159 | 55.4 ± 23.6 |
| **St. Paul, MN** | | | |
| AQS | 81, 331 | 9.1, 44.9 | 22.2 ± 14.0 |
| MESA Fixed | 63, 96 | 9.3, 35.7 | 22.2 ± 11.4 |
| MESA Home | 1, 5 | 2.4, 54.6 | 18.1 ± 8.7 |
| MESA Snapshot | 1, 3 | 5.0, 59.3 | 21.9 ± 10.4 |
| **Winston-Salem, NC** | | | |
| AQS | 306, 313 | 19.1, 22.4 | 20.7 ± 2.3 |
| MESA Fixed | 79, 100 | 9.8, 21.8 | 14.9 ± 5.4 |
| MESA Home | 1, 5 | 2.4, 34.3 | 10.4 ± 5.9 |
| MESA Snapshot | 1, 3 | 4.9, 95.3 | 21.5 ± 12.8 |

**Table S3.** Summary of LAC monitoring data.

| Region and site type | No. of observations per site Min, max | Site means ($10^{-5}$/m) Min, max | Site means ($10^{-5}$/m) Mean ± SD |
|---|---|---|---|
| **Baltimore, MD** | | | |
| MESA Fixed | 18, 92 | 0.42, 1.2 | 0.73 ± 0.30 |
| MESA Home | 1, 3 | 0.20, 1.76 | 0.62 ± 0.27 |
| **Chicago, IL** | | | |
| MESA Fixed | 4, 85 | 0.57, 0.91 | 0.76 ± 0.13 |
| MESA Home | 1, 4 | 0.27, 1.24 | 0.63 ± 0.17 |
| **Los Angeles, CA** | | | |
| MESA Fixed | 73, 82 | 0.95, 1.49 | 1.23 ± 0.23 |
| MESA Home | 1, 2 | 0.06, 2.87 | 1.27 ± 0.48 |
| **New York, NY** | | | |
| MESA Fixed | 49, 83 | 0.66, 1.65 | 1.18 ± 0.50 |
| MESA Home | 1, 3 | 0.06, 3.42 | 1.13 ± 0.53 |
| NYCCAS Reference | 50, 51 | 0.72, 1.14 | 0.93 ± 0.15 |
| NYCCAS Distributed | 6, 8 | 0.64, 2.75 | 1.23 ± 0.42 |
| **St. Paul, MN** | | | |
| MESA Fixed | 81, 91 | 0.39, 0.72 | 0.52 ± 0.17 |
| MESA Home | 1, 5 | 0.05, 0.99 | 0.43 ± 0.13 |
| **Winston-Salem, NC** | | | |
| MESA Fixed | 79, 92 | 0.44, 0.65 | 0.56 ± 0.09 |
| MESA Home | 1, 4 | 0.16, 0.86 | 0.47 ± 0.15 |

**Table S4.** Geographic covariates available for model development.

| Variable | Abbreviation | Buffer radii[a] | Source |
|---|---|---|---|
| **Roadway buffer variables** | | | |
| Total A1 road length (m) | A1 | 50m, 100m, 150m, 300m, 400m, 500m, 750m, 1km, 1.5km, 3km, 5km | TeleAtlas (2000) |
| Total A2 and A3 road length (m) | A2/A3 | 50m, 100m, 150m, 300m, 400m, 500m, 750m, 1km, 1.5km, 3km, 5km | TeleAtlas (2000) |
| Total A1, A1-A2, A1-A3, A2, A2-A3, and A3 intersections | Intersections | 500m, 1km, 3km | TeleAtlas (2000) |
| Total bus route length (New York only) (m) | Bus route | 50m, 100m, 150m, 300m, 400m, 500m, 750m, 1km, 1.5km, 3km, 5km | New York Department of Transportation (Quodomine R, personal communication) |
| Total truck route length (m) | Truck route | 50m, 100m, 150m, 300m, 400m, 500m, 750m, 1km, 1.5km, 3km, 5km, 10km, 15km | Bureau of Transportation Statistics (2009) |
| **Proximity variables[b,c] (distance in meters to nearest feature)** | | | |
| A1 road, A2 or A3 road, A1 or A2 or A3 road | Dist to road | N/A | TeleAtlas (2000) |
| Railroad; railyard; airport; large airport; coastline | Dist to feature | N/A | TeleAtlas (2000) |
| Truck route | Dist to feature | N/A | Bureau of Transportation Statistics (2009) |
| Bus route (New York only) | Dist to feature | N/A | New York Department of Transportation (Quodomine R, personal communication) |
| Commercial zone | Dist to feature | N/A | Price et al. (2006) |
| Small port; medium port; large port | Dist to feature | N/A | National Geospatial Intelligence Agency (2013) |
| Residual oil boiler; residual oil grade 6 boiler (New York only) | Dist to feature | N/A | Environmental Defense Fund (Silverman I, personal communication) |
| **Emission sources (tons/year)[c]** | | | |
| $NO_X$ emissions | $NO_X$ emissions | 3km, 15km, 30km | U.S. EPA Emission Inventory Group (2006) |
| $SO_2$ emissions | $SO_2$ emissions | 3km, 15km, 30km | U.S. EPA Emission Inventory Group (2006) |
| CO emissions | CO emissions | 3km, 15km, 30km | U.S. EPA Emission Inventory Group (2006) |
| $PM_{2.5}$ emissions | $PM_{2.5}$ emissions | 3km, 15km, 30km | U.S. EPA Emission Inventory Group (2006) |
| $PM_{10}$ emissions | $PM_{10}$ emissions | 3km, 15km, 30km | U.S. EPA Emission Inventory Group (2006) |
| **Residual oil** | | | |
| Residual oil boiler output (New York only) | Residual oil | 100m, 150m, 300m, 500m, 750m, 1.5km, 3km | Environmental Defense Fund (Silverman I, personal communication) |

| Variable | Abbreviation | Buffer radii[a] | Source |
|---|---|---|---|
| **Caline3QHCR**[c] | | | |
| Dispersion model out from Caline3QHCR model[d] | CALINE | 1.5km, 3km, 6km, 9km | (Eckhoff and Braverman 1995) |
| **Elevation** | | | |
| Elevation (m) | Elevation | N/A | National Elevation Dataset (USGS 2013) |
| **Census variables** | | | |
| Population density | Population | 500m, 1km, 1.5km, 2km, 2.5km, 3km, 5km, 10km, 15km | U.S. Census Bureau (2001) |
| **Land use variables** | | | |
| Percent of land use category: open water | LU: water | 50m, 100m, 150m, 300m, 400m, 500m, 750m, 1km, 3km, 5km | MRLC (2006) |
| Percent of land use category: developed open space | LU: open | 50m, 100m, 150m, 300m, 400m, 500m, 750m, 1km, 3km, 5km | MRLC (2006) |
| Percent of land use category: low intensity development | LU: low | 50m, 100m, 150m, 300m, 400m, 500m, 750m, 1km, 3km, 5km | MRLC (2006) |
| Percent of land use category: medium intensity development | LU: med | 50m, 100m, 150m, 300m, 400m, 500m, 750m, 1km, 3km, 5km | MRLC (2006) |
| Percent of land use category: high intensity development | LU: high | 50m, 100m, 150m, 300m, 400m, 500m, 750m, 1km, 3km, 5km | MRLC (2006) |
| Percent of land use category: deciduous forest | LU: forest | 50m, 100m, 150m, 300m, 400m, 500m, 750m, 1km, 3km, 5km | MRLC (2006) |
| Percent of land use category: woody wetland | LU: wetland | 50m, 100m, 150m, 300m, 400m, 500m, 750m, 1km, 3km, 5km | MRLC (2006) |
| Percent of land use category: ice and snow | LU: ice | 50m, 100m, 150m, 300m, 400m, 500m, 750m, 1km, 3km, 5km | MRLC (2006) |
| Percent of land use category: barren rock or sand | LU: barren | 50m, 100m, 150m, 300m, 400m, 500m, 750m, 1km, 3km, 5km | MRLC (2006) |
| Percent of land use category: evergreen forest | LU: evergreen | 50m, 100m, 150m, 300m, 400m, 500m, 750m, 1km, 3km, 5km | MRLC (2006) |
| Percent of land use category: mixed forest | LU: mix forest | 50m, 100m, 150m, 300m, 400m, 500m, 750m, 1km, 3km, 5km | MRLC (2006) |
| Percent of land use category: shrubland | LU: shrub | 50m, 100m, 150m, 300m, 400m, 500m, 750m, 1km, 3km, 5km | MRLC (2006) |
| Percent of land use category: grassland | LU: grass | 50m, 100m, 150m, 300m, 400m, 500m, 750m, 1km, 3km, 5km | MRLC (2006) |
| Percent of land use category: pastureland | LU: pasture | 50m, 100m, 150m, 300m, 400m, 500m, 750m, 1km, 3km, 5km | MRLC (2006) |

| Variable | Abbreviation | Buffer radii[a] | Source |
|---|---|---|---|
| Percent of land use category: cropland | LU: crop | 50m, 100m, 150m, 300m, 400m, 500m, 750m, 1km, 3km, 5km | MRLC (2006) |
| Percent of land use category: herbaceous wetland | LU: herb wetland | 50m, 100m, 150m, 300m, 400m, 500m, 750m, 1km, 3km, 5km | MRLC (2006) |
| Average impervious surface value | Imp surface | 50m, 100m, 150m, 300m, 400m, 500m, 750m, 1km, 3km, 5km | MRLC (2006) |
| 25th percentile of 2006 Normalized Difference Vegetation Index (NDVI) values | NDVI: Q25 | 250m, 500m, 1km, 2.5km, 5km, 7.5km, 10km | Carroll et al. (2008) |
| 50th percentile of 2006 NDVI values | NDVI: Q50 | 250m, 500m, 1km, 2.5km, 5km, 7.5km, 10km | Carroll et al. (2008) |
| 75th percentile of 2006 NDVI values | NDVI: Q75 | 250m, 500m, 1km, 2.5km, 5km, 7.5km, 10km | Carroll et al. (2008) |
| 50th percentile of 2006 Normalized Difference Vegetation Index (NDVI) values, April through September | NDVI: summer | 250m, 500m, 1km, 2.5km, 5km, 7.5km, 10km | Carroll et al. (2008) |
| 50th percentile of 2006 NDVI values, January through March and October through December | NDVI: winter | 250m, 500m, 1km, 2.5km, 5km, 7.5km, 10km | Carroll et al. (2008) |
| **Urban topography (New York and Chicago only)** | | | |
| Building height (floors) | Canyon metrics | N/A | New York City Dept. of City Planning (2004), City of Chicago (2011) |
| Distance-weighted mean of building heights on same and opposite side of street (floors) | Canyon metrics | N/A | New York City Dept. of City Planning (2004), City of Chicago (2011) |
| Block length (m) | Canyon metrics | N/A | New York City Dept. of City Planning (2004), City of Chicago (2011) |
| Indicator of being at block's end | Canyon metrics | N/A | New York City Dept. of City Planning (2004), City of Chicago (2011) |
| Indicator of no building on opposite side of street | Canyon metrics | N/A | New York City Dept. of City Planning (2004), City of Chicago (2011) |

[a]Measures were computed within circles of the given radii. [b]Truncated to between 10m and 25km, except for distance to residual oil boiler, which had an upper limit of 2km. [c]Covariate values were log-transformed. [d]At a small number of locations missing Caline3QHCR measures, values were imputed as the geometric mean of the values at AQS and fixed sites in that region.

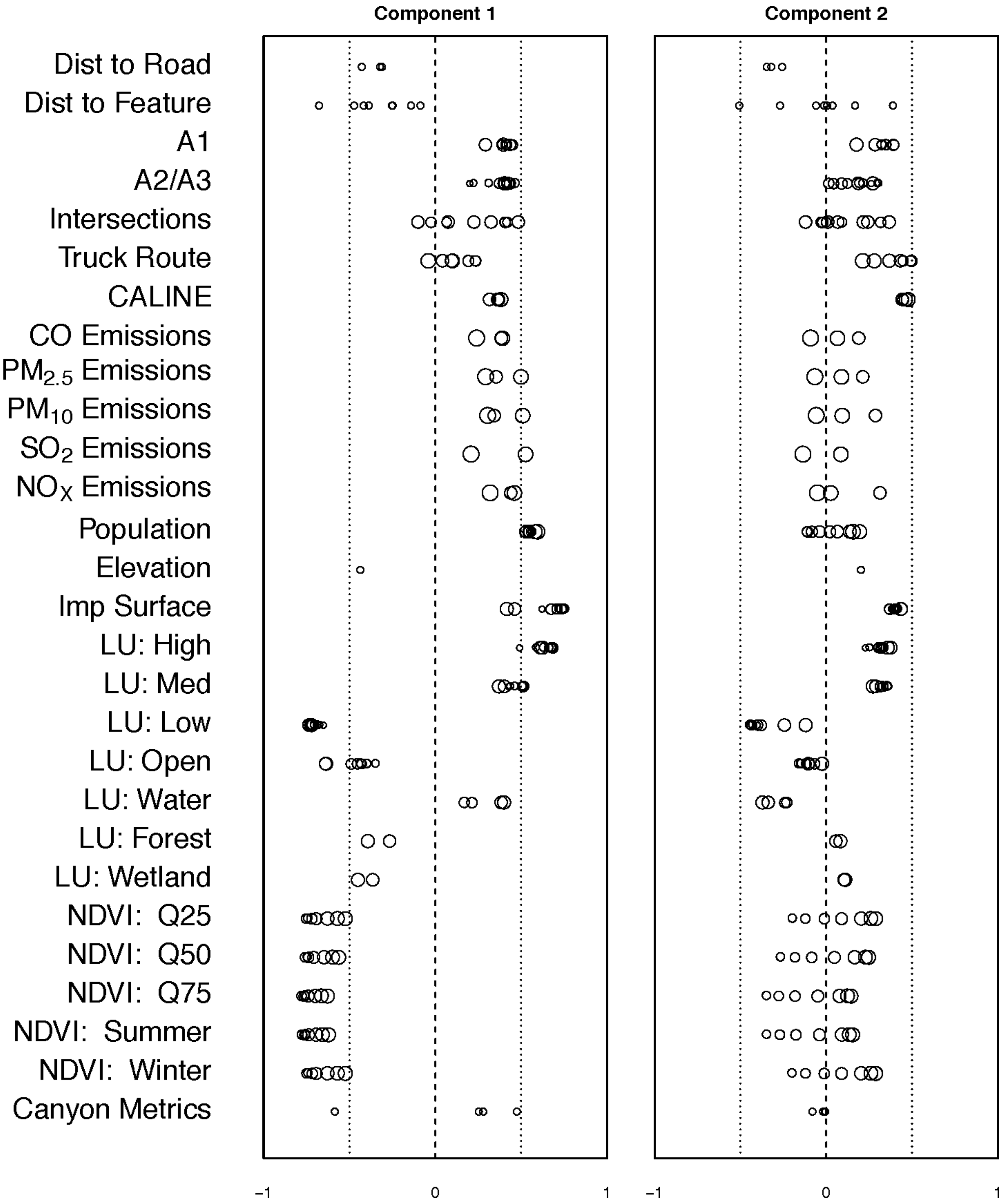


**Figure S1.** Correlations between geographic covariates and the two PLS components for the long-term average ($\boldsymbol{\beta_0}$) in the Chicago $NO_2$ model. Abbreviations are defined in Supplemental Material, Table S4. Different circle sizes represent different buffer radii.

**Table S5.** $R^2_{CV}$ for snapshot sites, by season. In Los Angeles, snapshot sites in Riverside were monitored at different times than the central Los Angles sites and are reported separately. Similarly, snapshot sites in New York City and Rockland County are reported separately.

| Region | Winter | Spring | Summer | Autumn |
|---|---|---|---|---|
| **$NO_2$** | | | | |
| Baltimore | 0.75 | – | 0.79 | 0.65 |
| Chicago | 0.56 | 0.53 | 0.53 | – |
| Los Angeles – Central | 0.49 | – | 0.55 | 0.60 |
| Los Angeles – Riverside | 0.44 | 0.41 | 0.43 | – |
| New York – NYC | 0.70 | 0.76 | 0.79 | – |
| New York – Rockland | 0.34 | 0.26 | 0.00 | – |
| St. Paul | 0.86 | 0.90 | 0.81 | – |
| Winston-Salem | 0.56 | – | 0.54 | 0.24 |
| **$NO_X$** | | | | |
| Baltimore | 0.63 | – | 0.61 | 0.72 |
| Chicago | 0.40 | 0.61 | 0.45 | – |
| Los Angeles – Central | 0.62 | – | 0.35 | 0.65 |
| Los Angeles – Riverside | 0.35 | 0.33 | 0.32 | – |
| New York – NYC | 0.51 | 0.43 | 0.37 | – |
| New York – Rockland | 0.17 | 0.09 | 0.03 | – |
| St. Paul | 0.79 | 0.81 | 0.70 | – |
| Winston-Salem | 0.41 | – | 0.34 | 0.47 |

**Table S6.** Leave-one-out cross-validation measures of predictive accuracy for two-week predictions at AQS and fixed sites. Units for RMSE are μg/m$^3$ ($PM_{2.5}$), ppb ($NO_2$ and $NO_X$), and $10^{-5}$/m (LAC).

| **Region** | **RMSE** | $R^2_{CV}$ |
|---|---|---|
| **$PM_{2.5}$** | | |
| Baltimore | 1.74 | 0.86 |
| Chicago | 2.02 | 0.80 |
| Los Angeles | 3.39 | 0.80 |
| New York[a] | 1.89 | 0.83 |
| St. Paul | 1.62 | 0.82 |
| Winston-Salem | 1.55 | 0.87 |
| **$NO_X$** | | |
| Baltimore | 6.95 | 0.88 |
| Chicago | 8.37 | 0.82 |
| Los Angeles | 12.6 | 0.86 |
| New York[a] | 12.4 | 0.67 |
| St. Paul | 4.72 | 0.86 |
| Winston-Salem | 6.76 | 0.63 |
| **$NO_2$** | | |
| Baltimore | 2.51 | 0.82 |
| Chicago | 3.77 | 0.71 |
| Los Angeles | 4.45 | 0.81 |
| New York[a] | 3.49 | 0.85 |
| St. Paul | 2.37 | 0.80 |
| Winston-Salem | 2.13 | 0.80 |
| **LAC** | | |
| Baltimore | 0.163 | 0.79 |
| Chicago | 0.117 | 0.68 |
| Los Angeles | 0.226 | 0.84 |
| New York[a] | 0.280 | 0.52 |
| St. Paul | 0.085 | 0.81 |
| Winston-Salem | 0.079 | 0.86 |

[a]Includes NYCCAS Reference Sites.

**Table S7.** Cross-validation measures of predictive accuracy for site means at monitoring locations in New York, from models fit without NYCCAS data. Leave-one-out cross-validation was used for AQS and fixed sites and ten-fold cross-validation was used for home sites. Units for RMSE are μg/m$^3$ ($PM_{2.5}$), ppb ($NO_2$ and $NO_X$), and $10^{-5}$/m (LAC).

| Pollutant | AQS and MESA Fixed: RMSE | AQS and MESA Fixed: $R^2_{CV}$ | AQS and MESA Fixed: $R^2_{CVreg}$ | MESA Home: RMSE | MESA Home: $R^2_{CV}$ | MESA Home: $R^2_{CVreg}$ |
|---|---|---|---|---|---|---|
| **$PM_{2.5}$** | 0.79 | 0.79 | 0.91 | 3.92 | 0.36 | 0.43 |
| **$NO_2$** | 1.69 | 0.94 | 0.95 | 3.88 | 0.77 | 0.77 |
| **$NO_X$** | 10.3 | 0.55 | 0.77 | 14.3 | 0.62 | 0.62 |
| **LAC** | 0.27 | 0.55 | 0.71 | 0.40 | 0.43 | 0.44 |

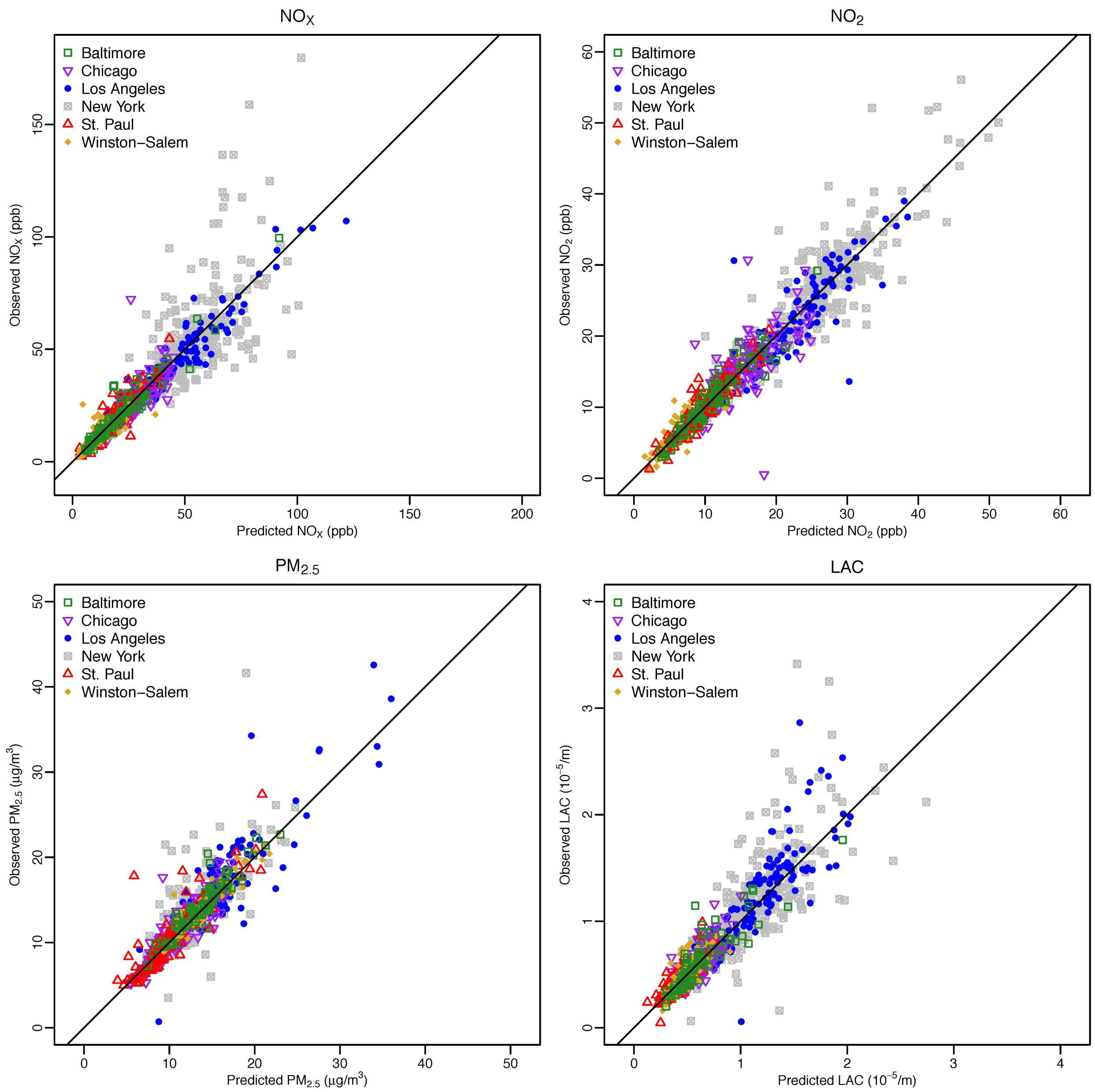


**Figure S2.** Cross-validated predictions and observations averaged over observed time period for home site monitoring locations for each pollutant. The New York data include NYCCAS distributed sites.

## References

Bureau of Transportation Statistics. 2009. National Transportation Atlas Database [digital data set]. Available: http://www.bts.gov/publications/national_transportation_atlas_database

Carroll ML, DiMiceli CM, Sohlberg RA, Townshend JRG. 2008. 250m MODIS Normalized Difference Vegetation Index [digital data set]. Available: http://glcf.umiacs.umd.edu/data/ndvi/

City of Chicago. 2011. Building Footprints. Available: https://data.cityofchicago.org/Buildings/Building-Footprints/w2v3-isjw

Eckhoff P, Braverman T. 1995. Addendum to the user's guide to CAL3QHC version 2.0 (CAL3QHCR user's guide).

MRLC: Multi-Resolution Land Characteristics Consortium. 2006. National Land Cover Database 2006 [digitial data set]. Available: http://www.mrlc.gov/nlcd2006.php

National Geospatial Intelligence Agency. 2013. World Port Index (Pub 150).

New York City Department of City Planning. 2004. New York City Primary Land Use Tax Lot Output (PLUTO) Property Characteristic Data. Available: http://www.nyc.gov/html/dcp/html/bytes/applbyte.shtml

Price CV, Nakagaki N, Hitt KJ, Clawges RC. 2006. Enhanced Historical Land-Use and Land-Cover Data Sets of the U.S. Geological Survey, U.S. Geological Survey Digital Data Series 240 [digital data set]. Available: http://pubs.usgs.gov/ds/2006/240

TeleAtlas. 2000. TeleAtlas Dynamap 2000 [CD_ROM].

U.S. Census Bureau. 2001. TIGER/Line Shapefiles. Available: http://arcdata.esri.com/data/tiger2000/tiger_download.cfm

U.S. EPA Emission Inventory Group. 2006. 2002 National Emissions Inventory Database [digital data set]. Available: http://www.epa.gov/ttn/chief/net/2002inventory.html

USGS. 2013. National Elevation Dataset. Available: http://seamless.usgs.gov/products/1arc.php
